# Evaluating Adverse Effects of Inhaled Nanoparticles by Realistic In Vitro Technology

**DOI:** 10.3390/nano7020049

**Published:** 2017-02-22

**Authors:** Marianne Geiser, Natalie Jeannet, Martin Fierz, Heinz Burtscher

**Affiliations:** 1 Institute of Anatomy, University of Bern, Baltzerstrasse 2, P.O. Box 922, 3012 Bern, Switzerland; natalie.jeannet@ifik.unibe.ch; 2 Institute of Aerosol and Sensor Technology, University of Applied Sciences and Arts Northwestern Switzerland, Klosterzelgstrasse 2, 5210 Windisch, Switzerland; martin.fierz@fhnw.ch

**Keywords:** 3R, aerosol, air-liquid interface, airway epithelia, electrostatic deposition, engineered nanoparticles, in vitro, NACIVT, toxicology, nanoparticles

## Abstract

The number of daily products containing nanoparticles (NP) is rapidly increasing. NP in powders, dispersions, or sprays are a yet unknown risk for incidental exposure, especially at workplaces during NP production and processing, and for consumers of any health status and age using NP containing sprays. We developed the nano aerosol chamber for in vitro toxicity (NACIVT), a portable instrument for realistic safety testing of inhaled NP in vitro and evaluated effects of silver (Ag) and carbon (C) NP—which belong to the most widely used nanomaterials—on normal and compromised airway epithelia. We review the development, physical performance, and suitability of NACIVT for short and long-term exposures with air-liquid interface (ALI) cell cultures in regard to the prerequisites of a realistic in vitro test system for inhalation toxicology and in comparison to other commercially available, well characterized systems. We also review doses applied to cell cultures in vitro and acknowledge that a single exposure to realistic doses of spark generated 20-nm Ag- or CNP results in small, similar cellular responses to both NP types and that cytokine release generally increased with increasing NP dose.

## 1. Introduction

Safe application of quickly growing nanotechnology requires a comprehensive clarification of adverse effects to humans and the environment. Nanoparticles in powders, dispersions, or sprays pose a yet undetermined risk for incidental exposure. Inhalation of engineered nanoparticles (ENP) in the processing industry and by consumer products is likely occurring. Thereby individuals with chronic pulmonary disease, as well as children and the elderly are expected to be more vulnerable than normal adult subjects [1,2,3,4]. Hence, safety testing of ENP requires including the susceptible population. In addition, efficient, economical, and ethically sound assessment of ENP toxicity needs animal-free test systems, mimicking realistic inhalation conditions.

Misleading responses of cells upon delivery of unrealistically high doses in terms of particle number and deposition time are very likely. Along these lines, pipetting particle suspensions on cells largely disregards basic physical laws of particle inhalation and deposition. Changes of particle characteristics upon their suspension in liquids are well known [5,6]. Particle exposure of cells at the air-liquid interface (ALI) has been shown to be superior for NP toxicity tests than exposure of submerged cells [6]. Recently, cell exposure systems have been developed delivering particles on cell cultures from a continuous aerosol flow, mostly by diffusional and/or gravitational deposition [7,8,9,10]. Few have applied electrostatic precipitation to increase the deposition efficiency [11,12,13,14]. In another system (ALICE), cloud settling combined with particle sedimentation as deposition mechanism has been used [15]. Currently, mainly commercially available systems have been adequately characterized in terms of their physical performance and suitability for cell exposures.

Within the framework of the Swiss National Research Program NRP 64 “Opportunities and Risks of Nanomaterials”, we developed a new instrument, the nano aerosol chamber for in vitro toxicity (NACIVT) [16] to expose cells to ENP out of a continuous air-stream, like within the living. The chamber is portable and the generation of aerosols is not an integral component of the chamber. Thus, toxicity assessments can be performed directly at the particle sources, e.g., at workplaces or on roadsides. Exposure experiments were performed with organotypic cell cultures replicating the human airway epithelium in health and disease [17]. We investigated whether and how nanoparticles affected epithelial structure and function, whether they initiated, inhibited, or aggravated (pro-)inflammatory responses, and we determined dose-response relationships. The newly developed system is unique as it fulfils all requirements for realistic testing in vitro: accurate aerosol deposition and cell cultures replicating the inner lung surface. The system is versatile, as it can be used for a wide range of particles, cell cultures, and particle sources. Moreover, it allows simultaneous exposure of up to 24 cell cultures to the same aerosol. The combined mechanical and biological unit advances the knowledge about possible health risks of inhaled ENP. In addition, this in vitro test system is a step forward in the development of alternative methods according to the 3R concept “reduce, replace, refine” in animal experimentation [18].

Several other deposition chambers for the same purpose have been introduced already, but only very few are fully characterized and commercially available. Among those are the devices by Cultex^®^ [7,13] and VITROCELL^®^ [19,20]. Their main characteristics will also be given in the following.

## 2. Airborne Nanoparticles of Concern

ENPs with a high risk for (incidental) inhalation exposure are those in form of suspensions, powders, or sprays and produced in large quantities [21,22]. Such nanomaterials are present in paints, textiles, or everyday consumer products, and their production is often powder based. Silver (Ag) and carbon (C) nanoparticles (NP) are of the most widely used materials in nanotechnology [22]. The antimicrobial activity of AgNP promoted their use in textiles, cosmetics, food packaging, implant coatings, wound dressings, and water disinfectants [23,24,25,26]. Thermal stability, electrical conductivity, UV protection, light weight, strength, and flexibility in surface functionalization boosted the application of CNPs in electronics, optics, paints, cosmetics, and medicine [27,28,29,30]. Thus, workplace safety is a major concern during nanoparticle production and processing. For consumers, products containing ENP in sprays are of particular concern. Persons of all age groups, gender, health status, and susceptibility to adverse effects of inhaled particles may be exposed to such ENPs. Furthermore, powder processing and using ENP-containing suspensions like sprays are known to generate significant concentrations of airborne NP [31,32]. In addition, hazards from exposure to NPs used as diagnostic tools or therapeutics in medicine [33,34] need to be assessed.

There are mainly two deposition mechanisms for airborne particles in the respiratory system: impaction and diffusion [35]. Impaction is efficient for coarse particles and leads to their deposition already in the nose, from where they can easily be removed again. The efficiency of diffusional deposition depends on the one hand on the diffusion coefficient, which increases with decreasing particle size and on the residence time, which increases deeper in the lung, where the flow velocity is small. Nanoparticles, for which impaction is inefficient, therefore, can penetrate deep into the lung and be deposited there [36].

The primary target tissue of deposited NP is the epithelium lining airways and alveoli. There has been consistent evidence from epidemiological and experimental air pollution studies that persons with pre-existing lung diseases like asthma, chronic obstructive pulmonary disease (COPD), and cystic fibrosis (CF) are more vulnerable to adverse effects of fine particulate matter (PM2.5, particles smaller than 2.5 µm in diameter) [1,2,3,4]. This emphasizes the necessity to include the susceptible population in ENP safety testing.

## 3. In Vitro Test System for Inhalation Toxicology

An in vitro system to assess adverse or beneficial effects of inhaled NP on the target tissue needs to mimic lung exposure with unprecedented accuracy: we need (i) an exposure chamber delivering NP as aerosols over time, in a quantitatively and qualitatively controlled and efficient way to the biological target; (ii) cell cultures replicating the morphology and function of the inner lung surface in health and disease; (iii) a set of relevant biomarkers to identify a variety of adverse effects, which are indicative for impaired lung homeostasis, i.e., initiation of pulmonary disease or interference with the course of disease; and (iv) the possibility for high-throughput toxicity screening.

### 3.1. Aerosol Generation

As the system is portable, it can be brought to the particle source. This may be particles in ambient air. Experiments have been carried out, for example, in Swiss and Asian cities (manuscripts to be submitted shortly).

Most test experiments were performed with carbon and silver particles. They were produced by a spark discharge generator (Palas, GFG1000, Karlsruhe, Germany). An electrical spark between two Ag or C electrodes volatilizes the material and leads to particle formation. Very high number concentrations of particles are produced leading to rapid agglomeration. In a subsequent tube furnace these agglomerates can be sintered to form more compact particles. Figure 1 in [37] shows the experimental setup in detail.

Any other particle source—e.g., combustion processes, spray produced particles, etc.—can be used. In our experiments, we additionally characterized the particles by measuring their size distribution with a scanning mobility particle sizer (SMPS) and on transmission electron microscopy (TEM) images. For electron microscopic analysis, particles were collected on TEM grids placed on a Transwell^®^ insert membrane (polyester membrane, 0.4 μm pore size; Corning, VWR, Dietikon, Switzerland) during 1 h of exposure in NACIVT [37,38].

### 3.2. Aerosol Deposition Chamber

**Figure 1:**
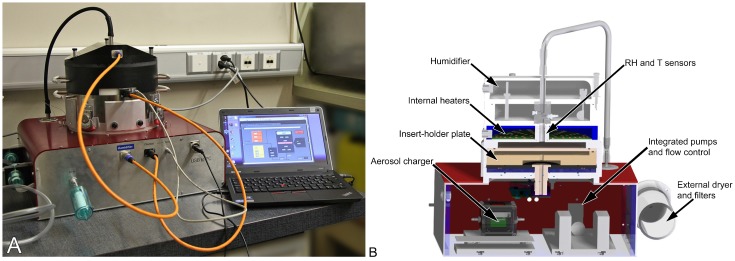
Nano Aerosol Chamber for In Vitro Toxicity (NACIVT). (**A**) Picture of chamber with dedicated laptop and LabVIEW software; (**B**) schematic section showing and describing the main parts of the chamber.

**Figure 2:**
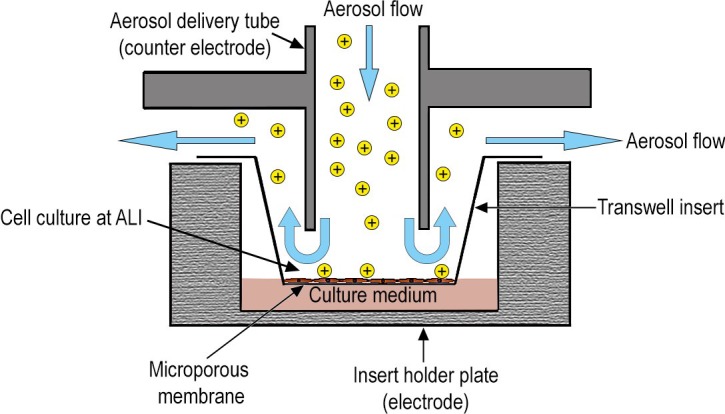
Particle delivery by electrostatic precipitation. Schematic section of an aerosol delivery tube, its Transwell^®^ insert and the insert-holder plate, demonstrating the aerosol flow and particle deposition on the cell culture. In this schematic, particles are previously charged by a unipolar diffusion charger.

In addition to delivering nanoparticles out of a continuous air-stream to the cell cultures, the instrument should mimic physiological conditions of inhaled particles in lungs as closely as possible. NACIVT [16,38] (Figure 1) was built in accordance with these requirements. The instrument allows the parallel deposition of particles on up to 24 cell cultures. To allow for use in field studies—i.e., at the particle source—particle generation by any means possible is outside the chamber. After entering NACIVT, the aerosolized particles are first charged (1–5 net charges per particle, depending on particle size) by a unipolar diffusion charger. Then, the particle containing gas is humidified to an adjustable relative humidity (RH, 85%–95%). The whole chamber is temperature controlled (usually to 37 °C). To efficiently deposit NP onto the cellular surface, particles are precipitated on the cells by electrostatic deposition (Figure 2), a well-known and recognized technique in aerosol science. All electrostatic precipitators are based on this technique; several devices to take samples for electron microscopy exist and have been used for a long time already [39,40,41,42]. In Fierz et al. [42], a detailed calibration for such a sampler is given. For measurements of particle concentration, the flow is split after the charger and a part passes through an aerosol electrometer.

**Figure 3:**
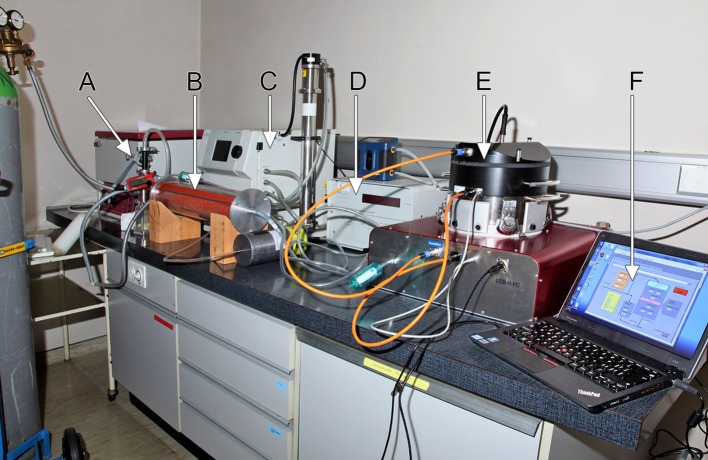
Experimental setup for deposition efficiency measurements with fluorescent polystyrene latex particles (PSL) [38]. (**A**) Nebulizer and (**B**) silica gel dryer for aerosol generation; (**C**,**D**) Instruments for SMPS—particle sizing; (**E**) NACIVT chamber for cell exposure at air-liquid interface; and (**F**) chamber-controlling laptop with a LabVIEW based program (National Instruments Switzerland GmbH, Ennetbaden, Switzerland).

NACIVT has been fully characterized, focusing on the physical performance of the chamber (Table 1). The tests performed evaluated temperature distribution and stability, performance of the humidity control, comparison of the precipitation efficiency in the different wells, distribution of the deposited particles on the Transwell^®^ inserts, as well as suitability for short and long term exposure experiments with ALI cell cultures [38]. For this purpose, aerosols of yellow green fluorescent polystyrene latex particles (PSL, 200 nm diameter; Fluoresbrite^®^, YG Microspheres, Polysciences, Warrington, PA, USA) and of silver nanoparticles (AgNP, 20 nm) were either deposited on cell-free Transwell^®^ inserts or on TEM grids containing inserts at an aerosol flow of 25 mL/insert/min. Figure 3 shows the setup for such an experiment. Particles were found to be evenly distributed on inserts. The number of deposited particles decreased towards the edge due to the gas flow pattern. This physical phenomenon, described by us previously [11,43], is inherent to all systems—though not investigated or not reported by others—and cannot be changed. For the TEM-sampler geometry, this topic is discussed in Fierz et al. [42]. Particle deposition efficiency, which depends on size, was 15% for 200-nm (PSL) and 40% for 20-nm (AgNP) particles. Electron microscopic analysis of particles collected on TEM grid showed solely single particles (PSL and AgNP), thus, particles did not agglomerate within the chamber [37,38]. Chamber compliance with cell exposures was evaluated using three cell models representing the primary target cell types of inhaled particles: fully differentiated human bronchial epithelial cells (HBE) with established ALI, primary porcine bronchoalveolar lavage (BAL) macrophages, and the human bronchial epithelial cell line BEAS-2B, which was mainly included for comparison with other in vitro studies. Cell cultures were exposed at the ALI to either particle-free (p-free) air or to aerosols of inert PSL particles for various lengths of time. Cytotoxicity was measured by the release of lactate dehydrogenase (LDH) from damaged cells at either 1 h (PSL) or 4 h (p-free) and at 24 h after exposure treatment. It was generally low and not different from unexposed control cells. Of further interest was testing whether deposited particles of different sizes—i.e., 200-nm PSL particles and 20-nm AgNP—reach the cell surface and are taken up by the cells. Laser scanning microscopy revealed PSL particles deposited on cells over the entire insert and within cells of both models tested (i.e., macrophages and BEAS-2B cells). As anticipated, primary porcine macrophages internalized PSL particle more efficiently than epithelial BEAS-2B cells. The spatial distribution of deposited AgNP (20 nm) was analyzed in HBE and BEAS-2B cell cultures by measuring the content of Ag in the apical lining layer and that associated with cells using microwave digestion followed by inductively coupled plasma mass spectrometry (ICP-MS) [44]. About one-third of Ag was located in the apical lining layer and two-thirds were associated with cells. Thus, we showed that deposited particles reach the cells on the entire surface of the Transwell^®^ insert and may be taken up by cells.

As already mentioned above, other chambers developed for the same purpose exist. In Table 2 some important properties of NACIVT and its competitors by Cultex^®^ (Cultex Laboratories GmbH Hannover, Germany) and VITROCELL^®^ (VITROCELL Systems GmbH, Waldkirch, Germany) are given. We solely considered chambers, which have been characterized with respect to the most important aerosol parameters, in particular the size dependent deposition efficiency and the spatial distribution of deposited particles on the cell cultures. Another important feature is how well the chambers mimic the physiological conditions in lungs. To our knowledge, no direct comparison of these chambers with cell tests has been published so far.

### 3.3. The Inner Lung Surface

The inner surface of the lungs functions as a physical, biochemical, and immunological barrier to separate outside from inside. Despite airways and alveoli varying considerably in size and cellular composition, as well as between species, the lung surface consists of the same basic structural elements. From the perspective of inhaled and deposited particles these are: (i) the liquid lining layer, which consists—from the alveoli to the trachea—of the surfactant film at the air-liquid interface and the aqueous phase beneath it; (ii) the mobile cells—i.e., in normal lungs mainly macrophages—which are fully immersed in the aqueous phase; and (iii) the highly differentiated, multicellular epithelium with its basal lamina [36]. Thus, it is these structures that the deposited particles will first interact with.

The conducting airways not only guide air to and from the alveoli [45], but they also keep the inhaled air humidified and warm, promote efficient precipitation of particles out of the gas stream, prevent epithelial damage by deposited particles, and transport deposited particles out of the lungs. This constant exposure to and interaction with deposited material of any type and its hazard potential requires high defense, repair, and regeneration capabilities of the epithelium (for review e.g., [46]). Moreover, the epithelium of the conducting airways is involved in most lung pathologies (for review e.g., [47]). Thus, to study adverse effects of inhaled particles in vitro, the target tissue of key importance is the respiratory epithelium.

To elucidate the features of particle-cell interactions in vitro, it is essential that cell cultures mimic the morphology and function of the inner lung surface in health and disease.

### 3.4. Airway Epithelia

To fulfil the different functions, the epithelium lining the conducting airways is a highly differentiated, polarized, multicellular, low-turnover epithelium with an established liquid lining layer at the ALI.

**Figure 4:**
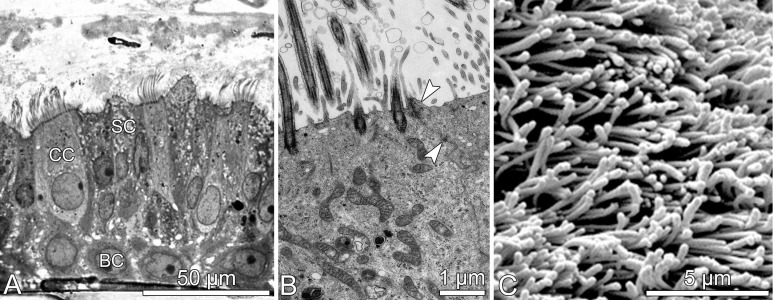
Morphology of in vitro differentiated human bronchial epithelia. (**A**) Light microscopic image exhibiting pseudostratified epithelium with basal (BC), ciliated (CC), and secretory (SC) cells; (**B**) transmission; and (**C**) scanning electron micrographs showing apical cell differentiations (cilia and microvilli) and junctional complexes (arrow heads in **B**).

Fully differentiated airway epithelia, as shown in Figure 4, may be derived from trachea and bronchi of human donors [17]. They exhibit the morphology and function of the native tissue. Morphology: (i) pseudostratified epithelium with ciliated, secretory and basal cells; (ii) junctional complexes—i.e., tight and adherent junctions, as well as desmosomes; (iii) basal lamina and hemi-desmosomes for anchorage. Functionality: (iv) ciliary beating and mucus production; (v) coordinated ciliary beating, thus, mucociliary transport; (vi) tight epithelium maintaining a permanent ALI; (vii) longevity (several months) and repair, i.e., replacement of cells lost from the epithelium. HBE may be derived from normal donors as well as from donors with pre-existing health conditions, i.e., CF, COPD (smokers), and asthma. Because HBE from donors with respiratory diseases differ from normal cells, they require characterization in regard to cell growth, differentiation, morphology, function, as well as baseline and maximal cytokine release prior to their use for experiments [37,48].

Cells used in the studies reported here were kindly provided by our collaborator M. Salathe. They were isolated from lungs obtained by the Life Alliance Organ Recovery Agency (LAORA) of the University of Miami and were available for research because the lungs were rejected for transplantation. IRB-approved consent for research with these tissues was obtained by LAORA and conformed to the declaration of Helsinki. The CF lung was donated by a transplant recipient and collected according to IRB-approved protocols.

Such advanced epithelial cell cultures contrast the often used proliferating, hardly differentiated, single-cell type cell lines, which are cultured submerged, and where apical medium is removed to mimic particle exposure at ALI. Moreover, responses to particle exposure have been shown to differ between HBE and cell lines (e.g., [37,48]).

### 3.5. Biological Endpoints

There are large numbers of biological tests available. We selected a set of biomarkers, which are indicative for impaired lung homeostasis, i.e., initiation of lung disease or interference with its course [37,48,49]. The measurements performed included qualitative as well as quantitative evaluation of epithelial morphology, function, and biochemical activity. Regular light microscopic inspection of the cell cultures allowed evaluating epithelial (i) differentiation to a functional respiratory epithelium; (ii) integrity and barrier function, i.e., maintenance of ALI; (iii) repair by basal cells, i.e., replacement of cells lost from the epithelium; and of (iv) mucociliary clearance by coordinated ciliary beating and directed transport of mucus. Additional electron microscopy provided insight into (v) the ultrastructure of cilia and junctional complexes, for example (Figure 4B). Supplementary biochemical and molecular analyses were performed to assess (vi) cytotoxic effects of aerosols resulting in cell necrosis and/or apoptosis by measuring LDH release and caspase-3 activity, respectively; (vii) effects on inflammatory processes by analysis of cytokines, primarily interleukin (IL)-6, IL-8, tumor necrosis factor alpha (TNF-α), and monocyte chemotactic protein 1 (MCP-1).

### 3.6. The Complete In Vitro System and Its Implementation

The mechanical NACIVT unit combined with advanced cell models like fully differentiated HBE derived from cells of proximal airways from normal and diseased donors, as well as appropriate analytics to assess biological responses provides a highly realistic in vitro system for safety testing of ENP. Moreover, NACIVT allows the study of aerosols with particle diameters up to micrometer size. Furthermore, aerosols can be generated by the method of choice, since this is not an integral part of the chamber. Because NACIVT is a portable, all-in-one system, it can be used directly at the particle source, i.e., in field experiments. The fully integrated design of NACIVT with stability of temperature and relative humidity also permits long-term (days, up to months) sub-acute exposures of cell cultures to aerosols. In addition, the longevity of re-differentiated airway epithelia (several months) permits repeated exposure to aerosols and evaluation of cell response over a longer time period after the exposure to aerosols. This is especially important, since most damage from inhaled (nano)particles occurs from (sub-)chronic exposure. The possibility to deposit particles on 24 cell cultures in parallel allows a very efficient operation. The on-line measurement dosing by the aerosol electrometer can also be used to determine the sampling time.

## 4. Effects of Selected, Commercial ENP on Normal and Diseased Airway Epithelia

Because of priority ranking in safety concern, we focused on exposure experiments with silver (Ag) and carbon (C) NP [37]. Quasi-spherical particles with an average mobile diameter of 20 nm were produced with a spark generator according to previously published protocols [50,51]. The particle size distribution was continuously measured with a scanning mobility particle sizer (SMPS). Normal and CF HBE, as well as BEAS-2B cells were exposed to three different NP doses (4 × 10^7^, 4 × 10^8^, or 4 × 10^9^ AgNP/cm^2^ or 3.5 × 10^8^, 3.5 × 10^9^, or 3.5 × 10^10^ CNP/cm^2^) for up to 1 h. Control cells were left untreated. Four hours after aerosol exposure, apical cell surfaces were washed with PBS to remove non-adherent NP. LDH and cytokine releases were assessed at 24 h, and apoptosis at 12 h after aerosol exposure.

CF HBE showed significantly increased LDH release, i.e., necrotic cell death after exposure to both NP types compared to unexposed CF controls (AgNP: *p* = 0.0003; CNP: *p* = 0.0011), normal HBE (AgNP: *p* < 0.0001; CNP: *p* = 0.0039), and BEAS 2B cells (AgNP: *p* < 0.0001; CNP: *p* = 0.0303) (Table 3 and Table 4). There were only subtle changes in caspase-3 activity observed after exposure to both NP types in any of the cell models. There was a general trend for increased release of IL-6, IL-8, and MCP-1 in all cell models after exposure to both NP types, except of MCP-1 in HBE, which appeared to be inhibited after exposure to AgNP. TNF-α was below detection limit in all cell cultures, particle types, and time points tested. There were no functional and structural alterations in cells observed after NP exposure.

In summary, the results indicate similar acute cellular responses after a single exposure to aerosols of 20-nm AgNP and CNP. There was a general trend for increased cytokine release with increasing NP doses. Substantial differences between HBE and BEAS-2B cells suggest primary cells are a more realistic model to study potential health effects of NP. Higher baseline levels of activated caspase 3 and secreted cytokines in CF than normal HBE may influence the course of CF lung disease and contribute to a decline in CF patients’ health. Our findings support epidemiological evidence that subjects with chronic airway diseases are more vulnerable to adverse effects of particulate air pollution. Thus, special consideration should be given to susceptible populations in toxicological studies of NP. In addition to epidemiological studies, our in vitro studies allow relating effects (biological response) directly to the cause (particle characteristics).

Overall, we found a single exposure to relevant doses of spark-generated AgNP and CNP to have low toxic effects on human bronchial epithelia. These findings are coherent with data from many rodent inhalation studies and a few studies in vitro showing no statistically significant cellular response after application of realistic NP doses [52,53,54,55,56,57]. Many other published in vitro studies, however, administered unrealistically high AgNP and CNP doses to achieve significant acute toxicity [58,59,60,61,62]. A typical dose of 50 µg/mL (equal to 5000 ng Ag/24-well insert) used in such studies is considerably higher than the NP doses applied in our study (0.58–58 ng Ag/24-well insert). Diverging NP toxicity may also result from different particle application techniques [63,64]. In our in vitro study and in inhalation experiments, NPs are delivered as aerosols to lung cells over an extended period of time, implying a low dose rate. That the mode of particle application and the related dose rate determine the magnitude of NP toxicity has been recently demonstrated in a study, where the inflammatory response was found to be higher after intratracheal instillation than after inhalation of the same NP dose [65]. Consequently, in vitro and in vivo studies with applications of particle suspensions likely overestimate NP toxicity.

## 5. Conclusions

We have developed and characterized a comprehensive system for realistic studies of inhaled particles in vitro. The system is robust, easy to use, and easily portable. It can be used in the laboratory, but also for field studies. The experimental data demonstrated that a single, short time exposure to realistic aerosols of AgNP and CNP induce moderate cytotoxicity and (pro-)inflammatory responses in airway epithelia, in a dose-response manner. The data obtained further indicate enhanced vulnerability of airway epithelia with pre-existing disease, in accordance with epidemiological studies on effects of air pollution. Our data further demonstrate significant discrepancies between primary (i.e., fully differentiated airway epithelia) and proliferating, single cell type bronchial epithelial cell lines. The differences were found to pertain to constitutively expressed fundamental parameters of airway disease as well as to biological parameters expressed in response to aerosol exposure.

The newly developed nano aerosol chamber for in vitro toxicity (NACIVT) is versatile, as it can be used for a wide range of particles, cell cultures and particle sources. Of further advantage is the parallel exposure of up to 24 cell cultures to the same aerosol. The comprehensive combined mechanical and biological unit advances the knowledge about possible health risk of inhaled (nano)particles. Meanwhile, NACIVT is used by renowned research groups in the USA (e.g., [66]), Canada, and Europe. These groups conduct research with various engineered/industrial as well as environmental (nano)particles using NACIVT. Moreover, NACIVT has passed testing for full mobility, as it has been successfully used in ambient air field studies in Asia and Europe. These world-wide studies will deliver additional characterization of the chamber and supplementary information on hazards, risks, and benefits of inhaled particles. Among the commercially available, well-characterized aerosol deposition chambers, NACIVT is the most compact and the easiest to transport.

The demand for aerosol deposition chambers mimicking realistic scenarios for hazard identification and toxicity assessments of inhaled (nano)particles in vitro is growing and will continue to increase in the future. Since there are already some well-characterized instruments available, validation and comparison of these chambers should be widely supported.

## Figures and Tables

**Table 1:** Characterization of the Nano Aerosol Chamber for In Vitro Toxicity (NACIVT) [38].

Parameter	PSL, 200 nm	AgNP, 20 nm	Cell Type
Aerosol conditioning			
Relative humidity * (%)	85–95	85–95	
Temperature * (°C)	37	37	
CO_2_ * (%)	5	5	
Aerosol flow per insert (mL/min)	25	25	
Particle distribution on Transwell^®^ inserts	Even, singlets	Even, singlets	No cells
Deposition efficiency (%)	15	40	No cells
Particle-cell contact			
CLSM	p-uptake	n.d.	Macs, BEAS-2B
ICP-MS	n.d.	^2^/_3_ assoc. with cells	HBE, BEAS-2B
Cytotoxicity ^#^ (%)			
Particles pipetted	<0.5	n.d.	BEAS-2B
P-free air	<0.5	n.d.	BEAS-2B
Exposed to aerosol	<0.5	n.d.	BEAS-2B

*: Adjustable and constant over time. PSL: polystyrene Latex particles, 200 nm in diameter, aerosol generation by nebulization; AgNP: silver nanoparticles, 20 nm in diameter, aerosol generation by spark ignition. ^#^: Measured at 24 h after one hour of treatment, % difference to unexposed control. CLSM: Confocal laser scanning microscopy; ICP-MS: Induction coupled plasma mass spectrometry; P-free: Particle free; P-uptake: Particle uptake; BEAS-2B: Human bronchial epithelial cell line; Macs: Macrophages; HBE: Human bronchial epithelia; n.d.: Not done.

**Table 2:** Physical performance of NACIVT in comparison to other aerosol deposition chambers for exposures of ALI cell cultures *.

Parameter	NACIVT [38]	Cultex^®^ RFS/RFS compact [7,13]	VITROCELL^®^ [19,20]
Cell exposure			
Number of cell cultures	24	3/6 in radial order around system inlet	6/12/24/48
Diameter of inserts (mm)	6.5	6.5/12/24/35 (Petri dish)	6.5/12/24/35 (Petri dish)
		special adapters	special adapters
Cell cultures separated from each other	Yes	Yes	Yes
Duration of exposures (h)	°24	°24	°24
Aerosol flow per insert (mL/min)	25, adjustable	5, 30, adjustable, separately for each chamber	2, 5, 100, adjustable, separately for each chamber
Temperature and control	On-line, temperature sensors within the chamber, adjustable from computer via LabVIEW	37 °C by temperature-controlled water flow (RFS)	37 °C by temperature-controlled water flow. Automatic temperature control by sensors
		On-line, temperature sensors within one chamber, adjustable (RFS Compact)	
Particle deposition			
Thermophoresis	No	No	Per extension kit
Electrostatic deposition	Switchable, bipolar, or unipolar charger	Can be added, unipolar charger	None
Electrical field	Up to 2 kV/insert, adjustable, both polarities DC or AC	40–450 kV/m, adjustable	±1.500 V, adjustable
Deposited dose	Aerosol electrometer, online	Gravimetric (precision balance)	Microbalance sensor, online photometer
Particle-free air control	Particle filter in-line before aerosol enters chamber	Parallel exposure of three inserts to test substance and three inserts to particle free air within one system	Independent clean air control modules or clean air positions in exposure module.
Concept of chamber	All-in-one	Modular	All-in-one or modular, automated exposure stations
Connectability to aerosol sources/generators	No restriction	No restriction	No restriction
Portable	Yes	Yes	Turnkey setups which can be moved to various locations

*: Exposure systems with continuous delivery of aerosols to cells and with declaration of physical performance were considered.

**Table 3:** Summary of changes in biomarkers in response to NP exposure showing the main contrast to the respective unexposed controls.

NP Type	Cell Model	LDH	Caspase-3	IL-6	IL-8	MCP-1	Epithelial Integrity
Ag	CF HBE	+	=	=	+	−	=
	Normal HBE	(−)	=	=	(+)	=	=
	BEAS-2B	−	=	=	=	=	=
C	CF HBE	+	=	=	=	=	=
	Normal HBE	=	=	+	+	(+)	=
	BEAS-2B	+ *	=	+	+	+	=

+: significant (*p* < 0.05) increase, in parenthesis not significant (*p* > 0.05). −: significant (*p* < 0.05) decrease, in parenthesis not significant (*p* > 0.05). = no response. *: values of control cultures were also high; NP: Nanoparticles; LDH: Lactate dehydrogenase; IL: Interleukin; MCP: Monocyte chemotactic protein; Ag: Silver; C: Carbon; CF: Cystic fibrosis; HBE: Human bronchial epithelia; BEAS-2B: Human bronchial epithelial cell line.

**Table 4:** Detailed data of changes in biomarkers in response to NP exposure.

NP Type	Cell Model	LDH %				Caspase-3 rfu/μg				IL-6 pg/mL				IL-8 pg/mL				MCP-1 pg/mL			
		Ctrl	D-1	D-2	D-3	Ctrl	D-1	D-2	D-3	Ctrl	D-1	D-2	D-3	Ctrl	D-1	D-2	D-3	Ctrl	D-1	D-2	D-3
Ag	CF HBE	8.7 4.7	23.1 ^#^ 7.7	18.2 ^#^ 5.2	14.4 ^#^ 6.3	46.9 18.7	85.0 ^#^ 14.2	34.1 4.4	54.4 ^#^ 3.8	134.3 66.8	118.4 28.0	155.4 54.8	189.3 104.9	4923 1752	8550 ^#^ 3232	7640 1404	9967 ^#^ 5582	19.6 6.3	16.0 5.4	11.8 4.4	11.6 ^#^ 6.4
	Normal HBE	17.6 4.1	14.1 3.7	14.9 3.9	11.7 ^#^ 3.6	20.7 8.2	39.6 21.7	18.7 1.6	13.1 4.1	29.2 23.2	36.1 15.7	29.5 12.4	50.9 23.0	4593 2263	6955 1773	5504 1665	7339 2397	4.5 3.0	2.4 0.8	2.9 1.6	2.0 1.9
	BEAS-2B	12.8 4.6	8.9 ^#^ 1.1	12.3 0.6	8.4^#^ 1.2	162.3 117.7	84.8 6.1	133.5 18.0	199.5 0.4	63.4 41.3	64.1 18.8	76.3 4.4	57.5 13.5	169.1 43.3	242.7 ^#^ 88.8	146.0 27.5	162.6 21.5	1298 301	1101 338	1573 78	1665 ^#^ 224
C	CF HBE	8.7 4.7	15.6 ^#^ 3.9	16.8 ^#^ 2.4	14.8 ^#^ 3.0	46.9 18.7	67.6 29.7	53.5 5.7	40.8 4.7	134.3 66.8	117.4 48.5	156.0 40.4	134.3 81.3	4923 1752	5651 1046	7394 2051	5017 1814	19.6 6.3	16.8 1.7	20.8 7.7	22.5 3.9
	Normal HBE	17.6 4.1	15.8 5.6	19.9 5.3	16.5 5.2	20.7 8.2	29.6 6.2	34.8 26.5	22.0 14.8	29.2 23.2	99.4 147.4	79.4 56.2	96.4 54.3	4593 2263	8571 9099	9992 ^#^ 3725	7697 1884	4.5 3.0	5.1 3.5	5.4 0.7	13.1 ^#^ 5.8
	BEAS-2B	12.8 4.6	12.0 1.1	12.0 1.4	25.7 ^#^ 4.8	162.3 117.7	127.1 31.3	90.0 22.8	393.4 ^#^ 29.4	63.4 41.3	159.5 ^#^ 25.9	129.8 ^#^ 37.3	151.9 ^#^ 8.7	169.1 43.3	246.4 ^#^ 66.2	221.3 ^#^ 10.5	225.7 ^#^ 23.4	1298 301	1791 ^#^ 123	1799 ^#^ 171	2005 ^#^ 226

Data are presented as mean values and SD. NP: Nanoparticles; LDH: Lactate dehydrogenase; IL: Interleukin; MCP: Monocyte chemotactic protein; Ag: Silver; C: Carbon; CF: Cystic fibrosis; HBE: Human bronchial epithelia; BEAS-2B: Human bronchial epithelial cell line. Ctrl: particle-free air control, i.e., no NP deposited. Deposited dose: Dose 1, D-1: 4 × 10^7^ AgNP/3.5 × 10^8^ CNP; Dose 2, D-1: 4 × 10^8^ AgNP/3.5 × 10^9^ CNP; Dose 3, D-3: 4 × 10^9^ AgNP/2 × 10^10^ CNP; ^#^ *p* < 0.05 to particle-free control.

## Abbreviations

Ag	silver
ALI	air-liquid interface
BAL	bronchoalveolar lavage
BEAS-2B	human bronchial epithelial cell line
C	carbon
COPD	chronic obstructive pulmonary disease
CF	cystic fibrosis
ENP	engineered nanoparticles
HBE	human bronchial epithelia
ICP-MS	inductively coupled plasma mass spectrometry
IL	interleukin
LAORA	Life Alliance Organ Recovery Agency
LDH	lactate dehydrogenase
MCP	monocyte chemotactic protein
NACIVT	Nano Aerosol Chamber for In Vitro Toxicity
NP	nanoparticles
p-free	particle-free
PM	particulate matter
PSL	polystyrene latex particles
RH	relative humidity
SMPS	scanning mobility particle sizer
TEM	transmission electron microscope/microscopy
TNF	tumor necrosis factor
